# The Importance of Amino-terminal pro-Brain Natriuretic Peptide Testing in Clinical Cardiology

**Published:** 2007-02-07

**Authors:** Van Kimmenade, Januzzi

**Affiliations:** 1 Department of Cardiology, University Hospital Maastricht, Maastricht, the Netherlands; 2 Department of Medicine, Massachusetts General Hospital and Harvard Medical School, Boston, Massachusetts, U.S.A

## Introduction

In 1981 de Bold et al. reported that the injection of atrial tissue extracts in rats induced natriuresis, a discovery that initiated a flurry of research leading to the discovery of a new class of cardiac hormones, the natriuretic peptides.^1^ These peptides represent the first line of response of the heart to defend the body against a number of processes, especially plasma volume expansion. Since circulating concentrations of natriuretic peptides are elevated in heart failure (HF) and given that clinical parameters for the evaluation and management of HF have a poor sensitivity and specificity, these peptides were a more than welcome addition to standard clinical evaluation, as a marker to identify HF. Nowadays, Brain Natriuretic Peptide (BNP) and its cleavage equivalent aminoterminal-proBNP (NT-proBNP) have proven its clinical diagnostic but also prognostic value in HF, and have shown to be superior as a diagnostic parameter for HF when compared to chest X-ray and parameters of physical examination.^2^–^4^ In this paper, we aim to discuss the background, the value, and of course the potential pitfalls encountered by clinicians using NT-proBNP testing in their daily clinical practice.

## The ABC of The Natriuretic Peptides: It’s All in The Family

**Figure 1:**
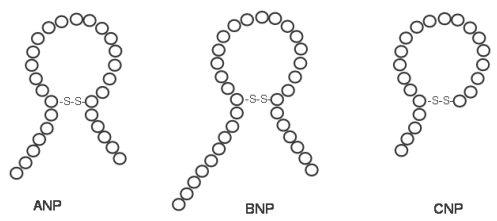
Structure of human cardiovascular natriuretic peptides: ANP, BNP and CNP.

The family of natriuretic peptides consists of several tightly-related members: atrial natriuretic peptide, B-type (formerly ‘brain’) natriuretic peptide, C-type natriuretic peptide and dendroaspis natriuretic peptide.^5^,^6^ Each of these peptides have the ability to induce natriuresis^7^ and share a common 17-aminoacid ring structure (Fig. 1).

Atrial natriuretic peptide (ANP) is primarily produced in both atria; production and release of ANP is typically triggered by increased atrial-wall tension typically following an increase of intravascular volume or pressure. The ventricles of normal adults produce only small amounts of ANP, but this production is enhanced in the ventricular tissue of fetuses/neonates and in hypertrophied ventricles.^1^,^5^,^8^

B-type natriuretic peptide (BNP), originally identified in the porcine brain, is found wherever myocardium is present, but is particularly concentrated in the cardiac ventricles. BNP and its cleavage equivalent NT-proBNP have emerged as superior markers for left-ventricular dysfunction, because when cardiomyocytes are stretched, these peptides are rapidly expressed at gene level and immediately released into the circulation. Importantly and in contrast to ANP, these peptides are also more stable,^9^,^10^ allowing for greater ease in their measurement. The similarities and differences between BNP and NT-proBNP will be discussed more into detail below.

C-type natriuretic peptide (CNP) has two forms, which are both products from a different cleavage process of a pro-CNP precursor. CNP predominates in the vascular endothelial cells, the central nervous system, the kidney and the pituitary gland. Plasma-concentration is low but tissue expression is high, suggesting a paracrine role for CNP.^11^,^12^ Dendroaspis natriuretic peptide (DNP) was isolated in the venom of the Green Mamba. Although it has proven to induce natriuresis, it remains unclear whether it plays any role in the physiology of mammals.^6^,^13^

## BNP and NT-proBNP: Brothers in Arms?

**Figure 2:**
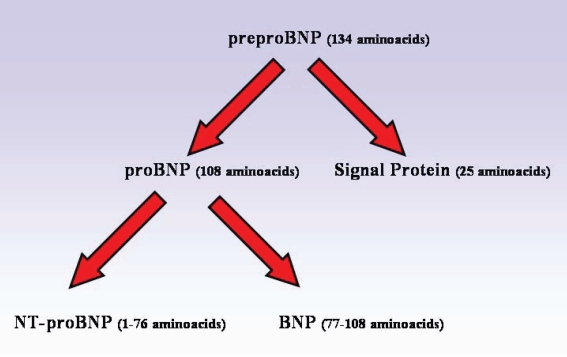
Derivation of BNP and NT-proBNP from their common predecessor proBNP_108_.

When cardiomyocytes are stretched, as in cardiac chamber overfilling, the BNP gene is quickly up-regulated, which leads to a rapid intracellular production of a 134 pre-pro-peptide. This pre-pro-peptide is rapidly cleaved to a 108 pro-peptide, proBNP_108_. This proBNP_108_ is enzymatically cleaved immediately in a 1:1 ratio to yield BNP (32 amino acids) and NT-proBNP (76 amino acids) which are directly released into the circulation.^14^ (Fig. 2) Interestingly, not all the proBNP produced in the cardiomyocytes is cleft since intact proBNP_108_ is measured in the circulation as well.^15^ Schellenberger et al. also report the presence of small amounts of glycosylated proBNP in the circulation, which is typically of much larger molecular weight.^16^ Athough proBNP_108_ is known to be detected by all BNP and NT-proBNP assays, whether glycosylated proBNP_108_ has any effect on conventional assays for natriuretic peptides is still not known.

While NT-proBNP is thought to be inert without any physiological function, BNP is biologically active and is responsible for several compensatory mechanisms in HF such as an increase of urine production and sodium excretion, a decrease of the aldosterone level, a lowering of the blood pressure and a lowering of the pulmonary capillary wedge pressure.^17^,^18^ BNP in physiological concentrations has too little effect to restore euvolemia but BNP is studied as a potential therapeutic agent in HF.^19^–^22^

Importantly, the clearance of the two peptides is different: BNP is cleared by several mechanisms, including the kidneys, specific clearance receptor-mediated degradation (natruretic peptide receptor type C; NPR-C) and catalytic degradation via enzymes, especially neutral endopeptidase 24.11,^23^ but possibly also by other enzymes.^24^ In contrast, NT-proBNP seems to be only renally cleared.^25^,^26^ These differences in clearances are responsible for the fact that BNP has a lower absolute plasma concentration and a shorter biological half-life of 20 minutes. As well, due to ongoing effects of neutral endopeptidases, BNP degradation continues even after phlebotomy. The ramifications of these facts were recently illustrated by Mueller and colleagues who demonstrated that after 2–4 months of storage in −20°C, less than 50% of BNP could be recovered in the plasma samples, while more than 90% of NT-proBNP was still intact;^27^ also a recent report demonstrates that measurable “BNP” is actually more likely a mixture of degraded BNP fragments, as highly-accurate gas chromatographic techniques showed no intact BNP in specimens with high readings for the marker.^28^ In contrast, NT-proBNP is a more stable molecule (with a biologic half-life of 60–120 minutes) with consequently absolute plasma concentrations of NT-proBNP that are 3–4 times higher than BNP, and without any special conditions required for sampling. In addition, it should be noticed that there is cross reactivity for NT-proBNP assays with proBNP_108_, but not with other BNP fragments.

Despite the potential differences and analytical concerns, recent head-to-head comparisons show that BNP correlates as well as NT-proBNP with clinical variables in patients with HF, indicating that most of the results of the BNP and NT-proBNP studies are generally transposable.^29^–^31^

## NT-proBNP: Diagnostic in Dyspnea

In the Emergency Department (ED), it is important to quickly diagnose the primary cause of acute dyspnea, since the success of most treatment therapies depends on the time-interval between presentation and treatment. Physicians therefore need a tool which is quickly available and adequately diagnoses or excludes HF. Although echocardiography can be performed quickly, it has to be carried out by an experienced person. Besides that, there is discussion what should be the echocardiographic gold standard for the diagnosis of diastolic HF.^32^,^33^

**Figure 3:**
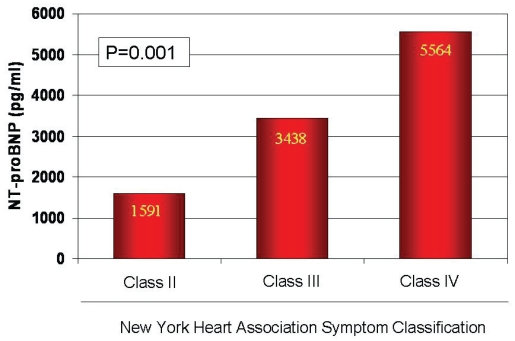
Median NT-proBNP concentrations as a function of dyspnea severity among 209 subjects with acute HF in the PRIDE study.

Smaller studies already had suggested that NT-proBNP was such a beneficial diagnostic tool in acute dyspnea.^31^,^34^ However, the ProBNP Investigation of Dyspnea in the Emergency Department (PRIDE) study was the landmark trial confirming that NT-proBNP measurement was valuable for the evaluation of dyspnea, representing a superior predictor of acute HF when compared to clinical assessment alone.^35^ This prospective, blinded trial of 600 dyspneic patients presenting to the ED, found that NT-proBNP concentrations were significantly higher in those with acute HF versus those without and demonstrated a close correlation to symptom severity. (Fig. 3) Besides, a subanalysis of this study also confirmed that NT-proBNP is not only diagnostic in systolic HF, but is also diagnostic in diastolic HF.^36^

Further insights to the value of NT-proBNP testing for the evaluation of acute dyspnea came from the International Collaborative Of NT-proBNP (ICON) Study.^4^ Briefly, the ICON Study consists of 1256 patients suffering from acute dyspnea, who presented to the ED of 4 university hospitals in Maastricht, Barcelona, Christchurch, and Boston. In the ICON population, 720 patients were finally diagnosed with acute HF as the primary cause of their shortness of breath while in the other 536 patients other causes for acute dyspnea were diagnosed. From these data, the authors derived the following optimal strategy for the implementation of NT-proBNP in acute dyspnea: for the **exclusion** of acute HF, a general age-independent cut-point of 300 pg/mL should be used, while for **diagnosis of** HF, age-dependent cut-points were more useful than a single cut-point: namely NT-proBNP > 450 pg/ml for patients < 50 years, > 900 pg/ml for patients in between 50 and 75 years, and NT-proBNP > 1800 for patients > 75 years. (Table 1)

This age-stratified approach was superior to using a single NT-proBNP cut-point; both were equally sensitive and specific, but by stratifying age, the positive predictive value of the marker increased significantly, speaking to the strong relationship between age and structural/functional physiologic changes with effects on natriuretic peptide values. Such an age-related effect on natriuretic peptides is by no means restricted to NT-proBNP; recent data suggest age is the most potent variable leading to an elevated BNP value in the absence of acute HF, with a 30% increase in the likelihood for a “false positive” BNP per decade of age.^37^ Accordingly, while a cut-point of 100 pg/ml is optimal for excluding HF in middle-aged patients, a wide “grey zone” (a zone between ‘rule out’ and ‘rule in’ cut-points, where predictive value is low) exists for BNP, with 500 pg/ml as a cut-point for including HF, and optimal BNP cut-points for younger or more elderly patients remain yet unknown. This age-dependent cut-point strategy proposed from ICON offers other advantages. Importantly, this age stratification narrows the “grey zone” considerably; this NT-proBNP cut-point strategy enables the physician to diagnose or exclude HF in 84% of the patients, without taking any other clinical or technical parameter into account.^38^ In contrast, for BNP testing, a single age-independent rule-in and and age-independent rule-out cut-point is advocated (i.e. BNP <100 pg/ml for excluding HF and BNP >500 pg/ml for diagnosing HF) but this strategy only succeeds in adequately confirming or rejecting the diagnosis of HF in 74% of the cases.^37^ The diagnoses of patients with grey zone NT-proBNP concentrations but not suffering from acute HF in the ICON studye are listed in Table 2.

## NT-proBNP: The Prognostic Peptide

**Figure 4:**
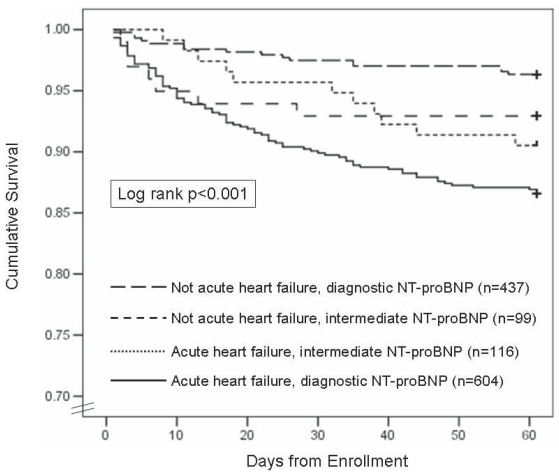
Kaplan-Meier curves demonstrating survival rates of all subjects in the ICON Study. ‘diagnostic’ means an NT-proBNP concentration above the rule-in cut-point for patients diagnosed with HF or below the rule-out cut-point in patients without HF.

Despite important improvements the last decade in the treatment of HF such as the introduction of beta-blockers or implantable defibrillators,^39^,^40^ the prognosis of patients with HF is still poor, with a 5 years survival of merely 50%.^41^ Furthermore, while the life-time risk of developing HF is 1 out of 5,^42^ paralleled by a substantial increase in costs for modern society,^43^ an accurate tool for stratifying risk in HF is worthwhile, in order to intervene more substantially with the hope to reduce such risk, and secondarily reduce the burden on the health care system. The classical predictors of prognosis in HF that are used in clinical practice typically include symptom severity, maximal oxygen consumption and left ventricular ejection fraction (LVEF), together with classical risk markers such as diabetes, older age and male gender.^44^–^47^ NT-proBNP has shown not only to be a powerful predictor of outcome in HF both on short term^48^ and one year time period,^49^ it is even a better predictor than maximal oxygen consumption, LVEF, or any other risk markers.^4^,^50^–^52^ Moreover, patients in whom NT-proBNP concentration decreases in reaction to therapy have a better prognosis;^53^ in an interesting pilot study including 69 patients, NT-proBNP was a superior guide for therapy than the combination of classical clinical symptoms and signs.^54^ Interestingly, the ICON study has shown that in 1256 patients with acute dyspnea, NT-proBNP was the best predictor of prognosis, regardless whether the patient was diagnosed with HF or not (Fig. 4) implying the importance of NT-proBNP for stratifying risk in dyspnea; the authors argue that in a dyspneic patient, there is no such thing as a “false positive” NT-proBNP, and elevations in a patient without acute HF should not be ignored.

## NT-proBNP: Special Considerations

### NT-proBNP and Valvular Heart Disease

Valvular heart disease is a common cause of HF, either by leading to volume overload (e.g. mitral regurgitation) or by causing pressure overload (e.g. aortic stenosis), and these relationships are frequently reflected in changes of NT-proBNP values. Since the clinical tools typically utilized to time the moment of intervention for valve repair or replacement are frequently limited to clinical judgment, an objective measure to guide the timing for intervention would be welcome, particularly since for instance recovery of LVEF or regression of LV mass is all but guaranteed after well-timed aortic valve replacement for aortic stenosis.^55^ Accordingly, the possible role of NT-proBNP in making the decision to operate upon these patients is of growing interest.

As an example, several studies have shown that NT-proBNP indeed correlates not only with onset and severity of symptoms but also with prognosis in aortic stenosis.^56^–^59^ Gerber et al. studied 74 patients with isolated aortic stenosis, and found that, after adjustment for age, sex, serum creatinine, aortic valve area and LVEF, that NT-proBNP concentrations were 1.74 times higher (1.12–2.69 95% CI) for symptomatic than asymptomatic patients with aortic stenosis.^57^ This is supported by the work of Weber and colleagues, who have also shown in their studies that NT-proBNP elevations were the earliest predictor of symptom onset, which is usually the moment when surgery is considered.^56^,^60^ Also in mitral valvular disease, several studies have indicated that the severity of valvular disease correlates with NT-proBNP concentrations, not only in mitral regurgitation,^61^–^63^ but also in mitral stenosis.^63^–^65^

While most of these studies predict a promising role for NT-proBNP in valvular heart disease management, further research is warranted in order to establish the role of NT-proBNP in a decision algorithm for valvular heart disease.

### NT-proBNP & Pulmonary Diseases

Patients with obstructive airway disease represent a particular challenge in the emergency department setting, as symptoms and signs of pulmonary disease may mimic (or mask) those of HF. Thus, the natriuretic peptides are a welcome advance for the correct identification of HF in patients with prior lung disease. In a recent study, Tung and colleagues have shown in 216 patients with underlying chronic obstructive pulmonary disease (COPD), that NT-proBNP was very useful for correctly identifying or excluding acute HF in this challenging patient population, superior to clinical judgment for this indication.^66^ Importantly, in this analysis, NT-proBNP also detected previously unsuspected HF in 22 patients with prior obstructive airway disease, an important diagnosis to make, in order to reduce the potential hazard associated with the dual diagnosis of pulmonary and cardiac insufficiency.

An important consideration in patients with pulmonary disease is involvement of the right ventricle as a consequence of their pulmonary disease: since NT-proBNP is produced everywhere cardiomyocytes are present it is well known that cardiomyocytes in the right ventricle produce NT-proBNP to a meaningful degree, particularly when stretched.^67^ Therefore, although the concentrations are usually lower, NT-proBNP can be elevated in with patients with increased right ventricular pressures, such as in pulmonary embolism (PE) or pulmonary arterial hypertension (PAH).^38^,^68^ In patients with PE, echocardiographic studies have confirmed that NT-proBNP concentrations correlate with echocardiographic and invasive measured parameters of right ventricular dysfunction.^68^–^70^ Also, Kucher et al. have shown that elevated concentrations of NT-proBNP correlate with adverse clinical outcome in patients with PE,^71^ which makes NT-proBNP not only a predictor of prognosis in these patients, but also a potential tool in therapy decision making (i.e. which patient should receive thrombolytic therapy).

In addition to acute right ventricular overload, NT-proBNP concentrations are also elevated in chronic PAH. Fijalkowska and colleagues demonstrated that NT-proBNP concentrations were not only increased in patients suffering from PAH, but also predicted 3-year death. Importantly, a high long-term mortality rate (61%) was observed in patients in whom plasma NT-proBNP levels increased by at least 50% during the follow-up period, while the mortality rate was significantly lower among other patients (12%; p< 0.001).^72^

An interesting study was performed by Goetze et al. in a population of patients evaluated for lung transplantation. While the patients with terminal parenchymal pulmonary diseases and normal LVEF had normal NT-proBNP concentrations (i.e. median 22 pg/mL; all below 300 pg/mL), patients with primary PAH had significantly higher NT-proBNP concentrations (median 906 pg/mL; p <0.001).^67^ Lastly, PAH can be caused by several mechanisms and is therefore sometimes considered as the next complication in systemic diseases such as systemic sclerosis, Mixed Connective Tissue Disease (MCTD), rheumatic arthritis etc. However, not all patients will develop PAH and it is difficult to predict which patients will develop PH and which not. NT-proBNP might play a role as a screening tool. Indeed, a small study by Allonore et al. performed in 40 patients with systemic sclerosis suggests that NT-proBNP can be used as a screening tool for the early stage of PAH, when clinical symptoms are not present yet.^73^

### NT-proBNP and Obesity

One important population where a marker for HF would be incrementally useful is in the obese patient. These patients do often have complaints of dyspnea but clinical parameters (e.g. elevated jugular veins, lower extremity edema) and technical investigations (chest X-ray and echocardiography) to make a diagnosis of HF are more difficult to interpret. Especially since both the prevalence of HF and obesity are increasing in our modern society, a sensitive marker for HF in the obese population would be most welcome.^43^,^74^

Surprisingly, HF patients with a higher body-mass index (BMI) have a better prognosis than patients with a normal BMI, the so-called ‘Obesity-paradox.’^75^–^78^ What is even more interesting, is that subjects with a higher BMI also have lower BNP concentrations^79^–^81^ which leads to speculation about the origin of this phenomena. For instance, it is known that BNP is also degraded by the NPR-C, which is abundantly expressed in adipocytes.^82^ Secondly, since it is more difficult to diagnose HF in obese subjects, it is possible that some patients were wrongfully diagnosed with HF, which obviously leads to a better prognosis.^83^,^84^

An important study in this perspective was performed by Krauser and colleagues.^85^ In this ancillary analysis of the PRIDE study, it was demonstrated that both BNP and NT-proBNP correlated negatively with BMI. This rejects the hypothesis that lower concentrations of BNP or NT-proBNP in obese subjects are caused by increased clearance in obesity since NT-proBNP is not cleared by adipocytes. This was confirmed by a small study that consecutively measured both NT-proBNP and BNP in subjects undergoing bariatric surgery. Following weight-loss surgery, postoperative BMI decreased dramatically in the study subjects, with parallel rise in both NT-proBNP and BNP over time.^86^ Importantly, BNP and NT-proBNP production in obese subjects is suppressed in the face of higher levels of ventricular wall stress than in non-obese patients. Thus, the lower values of both peptides do not reflect the obesity paradox in terms of prognosis, rather a derangement of neurohormonal regulation in obese HF patients.

The obvious ramifications of these data include this possibility of an obesity-related neurohormonal ‘suppression’ effect, such that the most obese patients may have a ‘handicap’ in their ability to secrete natriuretic peptides, and as such might be rendered more susceptible to hypertension, volume retention, and perhaps HF. As well, the interpretation of both BNP and NT-proBNP may be hindered by higher BMI; whether it interferes with the prognostic value of the markers remained unclear. However, recent data from the ICON study group have shown that despite a decreased production of NT-proBNP in obese subjects, NT-proBNP keeps its diagnostic capabilities across all BMI categories. (Antoni Bayes-Genis, in press).

### NT-proBNP and Renal Function: Is the clearance clear?

Despite all the recent experience with NT-proBNP testing, there is still some debate how to interpret NT-proBNP concentrations in patients with renal impairment, since there is a inverse correlation between NT-proBNP and glomerular filtration rate (GFR).^87^ Nevertheless, one should remember that there is a very tight relationship between cardiac and renal function. Indeed, the vast majority of patients with end stage renal disease (ESRD) have some degree of structural heart disease as well as plasma expansion related to decreased filtration. Conversely, the majority of the patients with HF have decreased renal function and risk factors for progressive renal disease.^88^,^89^ Therefore, a strong inter-relationship between NT-proBNP concentrations and parameters of renal function is in fact to be expected. This was confirmed by two studies examining the role of NT-proBNP testing patients with renal impairment;^90^,^91^ the germane question, however, is whether such a relationship will impair the utility of NT-proBNP testing in renal disease, and in these studies, NT-proBNP testing retained strong diagnostic and prognostic value even in the presence of renal impairment.

Considering the effects on renal disease and natriuretic peptides, certain very important concepts must be kept in mind. First, it is presumed that although steric and electrostatic factors also play a role in glomerular filtration, all molecules weighing less than 50 kDa should pass the glomerulus freely.^92^ Since both BNP (3.5 kDa) and NT-proBNP (8.5 kDa) weigh significantly less, filtration in the glomerulus cannot limit the clearance of both natriuretic peptides in patients with moderate renal impairment. Also, since renal impairment in HF mostly depends on decreased renal blood flow rather than a decrease in filtration in the glomeruli,^93^ it does not seem likely that the renal clearance of NT-proBNP is more hampered in renal impairment than the renal clearance of BNP. This also confirmed by the renal clearance studies of BNP and NT-proBNP in several population with moderate renal impairment.^94^–^96^ In addition, Tsutamoto and colleagues compared NT-proBNP and BNP production with serum concentrations by sampling in the aortic root and the coronary sinus, and they found that there was no decrease in clearance until GFR was <40 ml/min. Although there is a higher NT-proBNP/BNP ratio described in patients with renal impairment,^97^ one should bear in mind that BNP is also degraded by neutral endopeptidase 24.11 and the NPR-C receptor, which are both up-regulated in patients with renal impairment.^98^,^99^

Importantly, while observational analyses regarding the effects of renal disease on natriuretic peptide values are important, the real matter at hand is optimal application of these markers for clinical use, and the available data suggest that when used at optimal cut-points, NT-proBNP is powerfully diagnostic and prognostic in patients with renal disease and dyspnea.^4^,^90^ Indeed, when examining the available literature regarding BNP testing in renal disease in comparable populations,^100^ it is hard to find much of a difference between NT-proBNP and BNP testing in renal disease: both are affected, but both are valuable in these patients. Lastly, and very importantly emphasizing the importance of NT-proBNP in renal disease, the ICON study has also shown that both NT-proBNP and GFR are independent and additive predictors of prognosis in patients with HF.^101^ In this sub-study, all 720 patients in ICON diagnosed with HF were dichotomised according to median NT-proBNP concentration (4647 pg/mL) and according to presence or absence of National Kidney Foundation “moderate or more” renal impairment (i.e. GFR<60 ml/min/1.73m^2^). While both NT-proBNP > 4647 pg/ml and GFR <60 ml/min/1.73m^2^ were independent predictors of outcome (OR 2.67; 95% CI = 1.58–4.51 vs. OR 2.03; 95% CI = 1.18–3.49), the combination of a GFR<60ml/min/1.73m^2^ with a NT-proBNP >4647 pg/ml was the best predictor of 60 day mortality (OR 3.46; 95% CI = 2.13–5.63). Among subjects with a NT-proBNP above the median, those with a GFR <60 ml/min/1.73m^2^ had the worst prognosis while in subjects with a NT-proBNP below the median, prognosis was not influenced impaired renal function at presentation. (Fig. 4) These results reject the assertion that NT-proBNP is not useful in renal disease, and in fact is strongly prognostic in the presence of impaired renal function.

### NT-proBNP Testing in Ischemic Heart Disease

Since in coronary ischemia (including both stable and unstable angina as well as acute myocardial infarction [MI]), myocardial wall stress increases, and variable reductions in LVEF may also occur, the possible role of NT-proBNP testing for evaluation of ischemic heart disease has also been studied.^102^,^103^ Seminal studies found indeed that NT-proBNP was a potential useful diagnostic and prognostic marker in MI.^104^,^105^ Jernberg and colleagues also found in 775 patients admitted for acute chest pain without ST elevations that the patients in the 2nd, 3rd, and 4th quartile had a relative risk of subsequent death of 4.2, 10.7 and 26.6 when compared to the lowest quartile.^106^ Several studies have confirmed that NT-proBNP is an independent predictor of mortality in patients suffering from an acute coronary syndrome (ACS) and moreover, even a stronger predictor for mortality than troponin testing in this setting, regardless of any signs of HF.^105^,^107^–^110^

These findings did not perfectly fit in the concept that NT-proBNP is merely a marker of acute myocardial stretch and suggest another mechanism. Firstly, NT-proBNP is associated with older age, hypertension, renal impairment, diabetes mellitus and pre-existent left ventricular dysfunction, which are also predictors of worse outcome. It might therefore be that NT-proBNP results “sum” up all these parameters, and represent a “final common pathway” marker for prognosis. However, when corrected for these factors in multivariate analysis, NT-proBNP remains an independent predictor.^108^ Secondly, other studies found elevations of BNP correlate with the size of ischemic myocardium, which is not necessarily stretch related;^111^,^112^ as well, recent studies from the stress testing literature show unequivocal evidence for elevations in both NT-proBNP and BNP in patients with ischemia but with normal LV function.^113^,^114^ Finally, Goetze et al. found in vitro, that the secretion of NT-proBNP in ischemia is mediated by a different pathway, triggered by hypoxia rather than myocardial stretch.^115^,^116^ Accordingly, in addition to valuable risk stratification in identifying those with an ACS on top of a dysfunctional ventricle, NT-proBNP levels may also reflect degree and extent of myocardial ischemia. The role of NT-proBNP for routine inclusion in evaluation of chest pain is now underway.

## Conclusions

NT-proBNP has now become an established marker in modern medicine, thanks to the broad array of data supporting its role in a number of disease states, most notably being HF. The results of NT-proBNP testing are quickly available and have a high diagnostic value, even superior to single clinical parameters and chest X-ray. This diagnostic accuracy remains also in the presence of moderate renal impairment, underlying pulmonary disease or obesity. Besides its high diagnostic value, it is more important to realize that NT-proBNP is also a most strong predictor of prognosis, not only in HF, but also in valvular heart diseases, pulmonary hypertension, and ACS. Thus, while troponins are necrosis markers and could be considered ‘the epitaph’ of the cardiomyocyte, NT-proBNP should be considered an ‘S.O.S. signal,’ which indicates that the cardiomyocyte is distress and some kind of intervention is needed.

The future of NT-proBNP testing is bright: at present numerous studies are examining the role of NT-proBNP testing for “screening” of apparently well patients in primary are offices for incipient HF, while the role of NT-proBNP testing for the monitoring and titration of outpatient HF therapy is also under close examination.

## Figures and Tables

**Figure 5:**
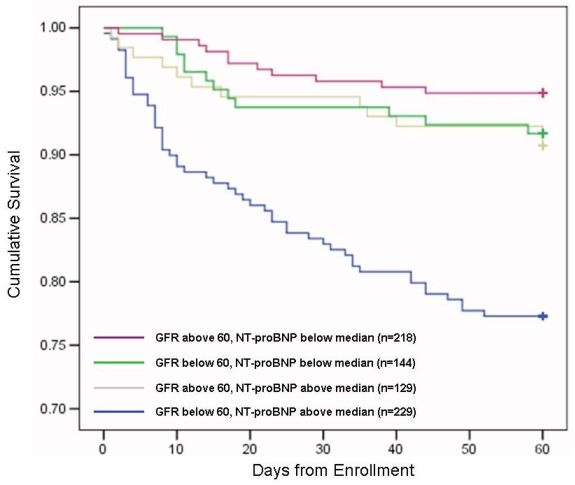
Survival curves of HF subjects in ICON as a function of GFR and NT-proBNP concentration on admission (log-rank p<0.001).

**Table 1:** Optimal NT-proBNP cut-points for the diagnosis or exclusion of acute HF among dyspneic patients.

Category	Optimal cut-point	Sensitivity	Specificity	PPV	NPV	Accuracy
**Confirmatory (“rule in”) cut-points**						
**<50 years (n = 183)**	450 pg/ml	97%	93%	76%	99%	94%
**50–75 years (n = 554)**	900 pg/ml	89%	81%	82%	88%	85%
**>75 years (n = 519)**	1800 pg/ml	85%	72%	92%	55%	83%
**Rule in, overall**		**87%**	**83%**	**86%**	**66%**	**85%**

**Exclusionary (“rule out”) cut-point**						
All patients (n = 1256)	300 pg/ml	99%	60%	77%	98%	83%

**Table 2:** Diagnoses in patients from the ICON studye with a “grey zone” NT-proBNP, not suffering from acute HF.

Diagnosis	Patients (n = 99)			
Chronic Obstructive Pulmonary Disease/Asthma				12%
Pneumonia/bronchitis			12%	
Acute Coronary Syndrome/chest pain				12%
Arrhythmia/Bradycardia			8%	
Lung cancer (including metastases)				5%
Anxiety Disorder		5%		
Pulmonary Emboli		3%		
Pulmonary hypertension			1%	
Pericarditis	1%			
Other*	21%			
Unknown	19%			

* Includes: anemia, cancer, gastrointestinal pathologies, sleep apnea, septic shock.
